# Clinicopathological study of ophthalmic cutaneous and mucocutaneous non-langerhans cell histiocytic lesions

**DOI:** 10.1186/s12886-024-03388-8

**Published:** 2024-03-19

**Authors:** Hind Manaa Alkatan, Dalal R. Fatani, Azza M.Y. Maktabi, Tariq A. Alzahem

**Affiliations:** 1 Ophthalmology Department, College of Medicine, King Saud University, P.O. Box 266, Riyadh, 11362 Saudi Arabia; 2 Pathology and Laboratory Medicine Department, College of Medicine, King Saud University, Riyadh, Saudi Arabia; 3 King Saud University Medical City, King Saud University, Riyadh, Saudi Arabia; 4 Oculoplasty and Orbit Surgery, King Khaled Eye Specialist Hospital, Riyadh, Saudi Arabia; 5 Pathology and Laboratory Medicine Department, King Khaled Eye Specialist Hospital, Riyadh, Saudi Arabia

**Keywords:** Histiocytes, Non-langerhans, Eyelid, Iris, Choroid, Xanthogranuloma, Hyphema, Juvenile

## Abstract

**Background:**

The “C group” of the histiocytic disorders is characterized by non-Langerhans-cell histiocytic lesions in the skin, mucosal surfaces, or both, out of which Juvenile xanthogranuloma (JXG) is the most common typically affecting the skin. The eye is the most common extra-cutaneous site of JXG., we aim at providing our clinical and histopathological experience with this group of diseases including the adult-onset xanthogranuloma (AXG).

**Methods:**

This is a retrospective cohort study of all patients with the tissue diagnosis of ocular and periocular cutaneous and mucocutaneous non-LCH disorders who presented to us over a period of 25 years (January 1993 to December 2018).

**Results:**

Twenty patients were diagnosed as “Group C” disease with an age range of 2 months-60.9 years. Eleven patients were females (55%) and nine were males (45%). The involvement was mostly unilateral in 80.9%. All cases fell into the xanthogranuloma family with 11 JXG patients, 8 AXG patients of skin and ocular surface, and one patient with solitary reticulohistiocytoma (SRH). The clinical site of involvement in JXG was primarily in the eyelid in 5 patients (45%), ocular surface lesions in 2 (18%), iris in 2 (18%), choroidal and bilateral orbital lesions in 1 patient each (9%). The group of AXG, presented equally with eyelid lesions in 4/8 and ocular surface lesions in 4/8. The non-Langerhans’ histiocytic infiltrate showed supportive immunohistochemical staining properties (reactive to CD68 marker and negative to S-100 and langerin markers).

**Conclusion:**

Among the rare histiocytic disorders, xanthogranulomatosis is the commonest and has wide clinical manifestations. Accurate diagnosis needs to be supported by typical histopathological findings. JXG was the commonest in our study with relatively older mean age at presentation and frequent eyelid rather than iris involvement. AXG is often confused with xanthelasma when involving the eyelids with corneal limbal involvement is relatively frequent.

## Introduction

Histiocytic disorders are generally rare, and their classification has been evolving into 5 major groups due to their wide variation of clinical manifestations, molecular pathology, and the cell of origin [1, 2] One of these is the “C group”, which includes variable non-systemic non-Langerhans cell histiocytosis (LCH) xanthogranulomatous disorders involving the skin, mucosal surfaces, or both [3]. The hallmark of this cutaneous and mucocutaneous histiocytosis is the proliferation and accumulation of histiocytes in the skin and mucocutaneous tissues clinically presenting as solitary nodules or in the form of multisystemic involvement with variable anatomic localization and disease severity resulting in diagnostic challenges [3]. Rarely, C group diseases can involve ophthalmic structures including eyelids, ocular surface, intraocular tissues, and orbit [3, 4].

The most common C group diseases are juvenile xanthogranuloma (JXG) followed by adult xanthogranuloma (AXG). JXG is commonly a benign cutaneous disease manifesting with orange skin lesions seen in young patients in the head and neck areas. However, it can involve the central nervous system (CNS), lungs, liver, spleen, and other visceral locations [5, 6]. Ocular involvement has been described in 0.3 to 10% of children with cutaneous JXG [7]. Adult xanthogranuloma (AXG), on the other hand, is typically an isolated xanthogranulomatous disease with similar clinical and histopathological presentation to JXG [8]. IgG4-related disease (IgG4-RD), which is caused by infiltration of multiple organs by IgG4 + plasma cells, may progress to IgG4-related ophthalmic disease (IgG4-ROD) and has been reported in relation to several histiocytic diseases such as AXG [9, 10]. Our review focuses on the cutaneous non-LCH under the xanthogranuloma family in particular JXC, AXG and solitary reticulohistiocytoma.

## Methods

This is a retrospective study conducted at King Khaled Eye Specialist Hospital (KKESH) and King Abdulaziz University Hospital (KAUH) in Riyadh, Saudi Arabia after being approved by the Human Ethics Committee/Institutional Review Board (HEC/IRB) at KKESH with a collaborative agreement between the 2 hospitals. Patients were recruited based on the histopathological data base with the diagnosis of mucocutaneous xanthogranulomatosis during the period January 1993 to December 2022. The corresponding demographic, clinical data, radiology findings, systemic treatment, and follow up were collected via medical records review using a specially designed data collection sheet. Histopathological review of all cases for further confirmation of the diagnosis was conducted by a team of two ocular pathologists. Descriptive analysis was performed where is applicable in addition to simple data analysis using SPSS version 22.0 (IBM Inc., Chicago, Illinois, USA) to represent the results in the form of numbers and percentages. Literature review of this group of xanthogranulomatous disorders was carried on using MEDLINE for English language-written studies.

## Results

We included 20 patients with the final tissue diagnosis of mucocutaneous xanthogranuloma with an age range of 2 months to 60.9 years, mean age of 21 years, and a median age of 6.79 years at presentation. No significant gender disparity was noted as 55% were females (*n* = 11) and 45% were males (*n* = 9). The involvement was mostly unilateral in 80.9%. Eyelids were primarily involved in 9 patients (45%), followed by ocular surface (conjunctival/limbal) lesions in 6 patients (30%), iris lesions in 2 patients (10%), and 1 patient with choroidal involvement in one eye and bilateral orbital lesions. Two out of 20 patients (10%) were noted to have multiple histiocytic lesions. None of the patients had other lesions in visceral locations representing major systemic involvement. The presenting clinical features included eyelid swelling (45%), ocular surface irritation (15%), decreased vision (10%), eyelid erythema (10%), orbital pain (10%) and hyphema (10%). The median duration of symptoms was 1.5 years (range 1 week-7 years).

The lesions were analyzed irrespective of the lesion’s location based on the classification of the xanthogranuloma group into 11 patients with JXG, 8 patients with AXG and 1 patient with solitary reticulohistiocytoma (SRH).

The JXG group included 11 patients (55%) summarized in Table 1. The age in this group ranged from 2 months − 17 years with a mean of 4.5 years and a median of 4.9 years. There was no significant gender predilection with female to male ratio of 1.2:1.

**Table 1:** Demographics of 11 patients with ophthalmic JXG according to the primary site of involvement

Variables	Eyelid (5 patients)	Orbit (1 patient)	Conjunctiva/limbus (2 patients)	Iris (2 patients)	Choroid (1 patient)	Total (11 patients)
**Age, years**						
Median	4 years	5.9 years	9 years	2.5 months	6 years	4.9 years
**Gender, number**						
Male	3	1	1	0	0	5
Female	2	0	1	2	1	6
**Laterality**						
Unilateral	4	0	2	1	1	8
Bilateral	1	1	0	1	0	3
**Clinical presentation**	5 Eyelid swelling/mass, 1 lagophthalmos	1 proptosis	2 Nodule/ swelling over the eye	2 Hyphema	1 decreased vision	-
**Associated lesions**	1 patient had facial and trunk lesions	N/A	N/A	Occipital scalp, eyelid, face, and trunk lesions	N/A	-
**Duration of symptoms**	(2 weeks – 4 years) Median: 1.3 years	8 weeks	1 year	2 weeks	3 weeks	(2 weeks – 4 years) Median: 8 weeks

**Fig. 1:**
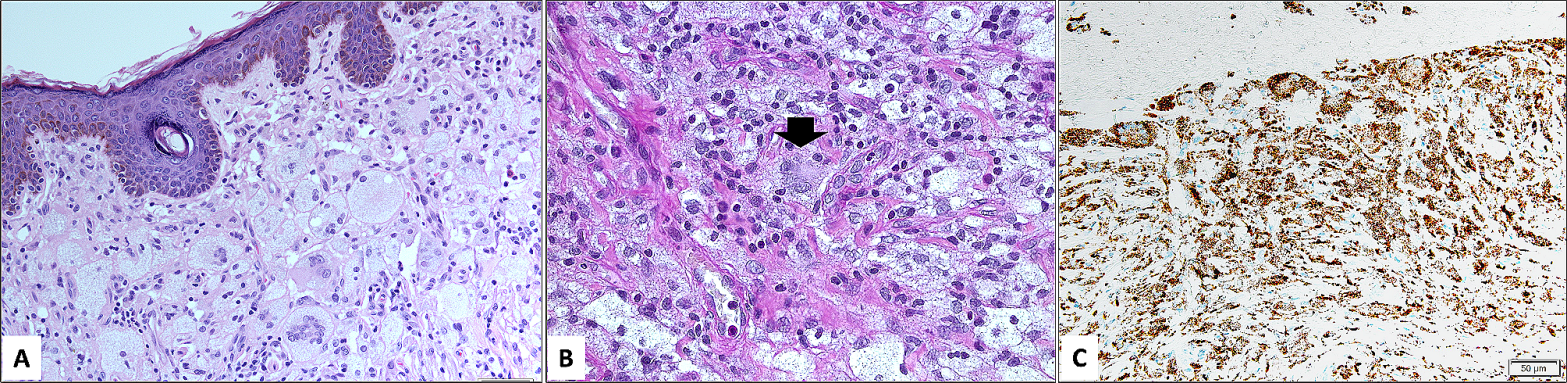
**A** & **B**: The histopathological appearance of the foamy histiocytes and typical Touton giant cell (black arrowhead) in a case with cutaneous eyelid juvenile xanthogranuloma (Original magnification x 200 in A and x 400 in B, Hematoxylin and eosin). **C**: The expression of the non-Langerhans histiocytes using immunohistochemical staining (Original magnification x 200 CD-68)

The eyelid lesions were mostly circumscribed histopathologically and showed dense infiltration by foamy histiocytes within the dermis with Touton giant cells (Fig. 1A & B). The histiocytic infiltrate was reactive to CD-68 histiocytic marker (Fig. 1C).

Two patients presented with isolated unilateral conjunctival/limbal lesions with similar histopathological features.

Iris JXG cases involved patients in their early infancy with unilateral or bilateral hyphema Table 1. Biopsies from associated skin lesions confirmed the diagnosis of JXG. The treatment, recurrence, and follow-up for JXG cases is summarized in Table 2.

**Table 2:** Summary of the management and outcomes of patients with ophthalmic JXG

Variables	Eyelid (5 patients)	Orbit (1 patient)	Conjunctiva/limbus (2 patients)	Iris (3 patient)	Choroid (1 patient)	Overall (11 patients)
**Therapy/intervention, number**						
Surgical ^b^	5	1	2	3	1	11
Medical ^c^	0	0	0	3	0	3
**Duration of follow-up, months**						
Median	4.5 years	15 years	6 months	1 year	9 months	14.5 months
**Recurrence/progression, number**						
Absent	5	1	2	3	1	11

^b^ Includes excisional biopsies (single or multiple), debulking procedures, and enucleation

^c^ Includes topical steroids and atropine

**Fig. 2:**
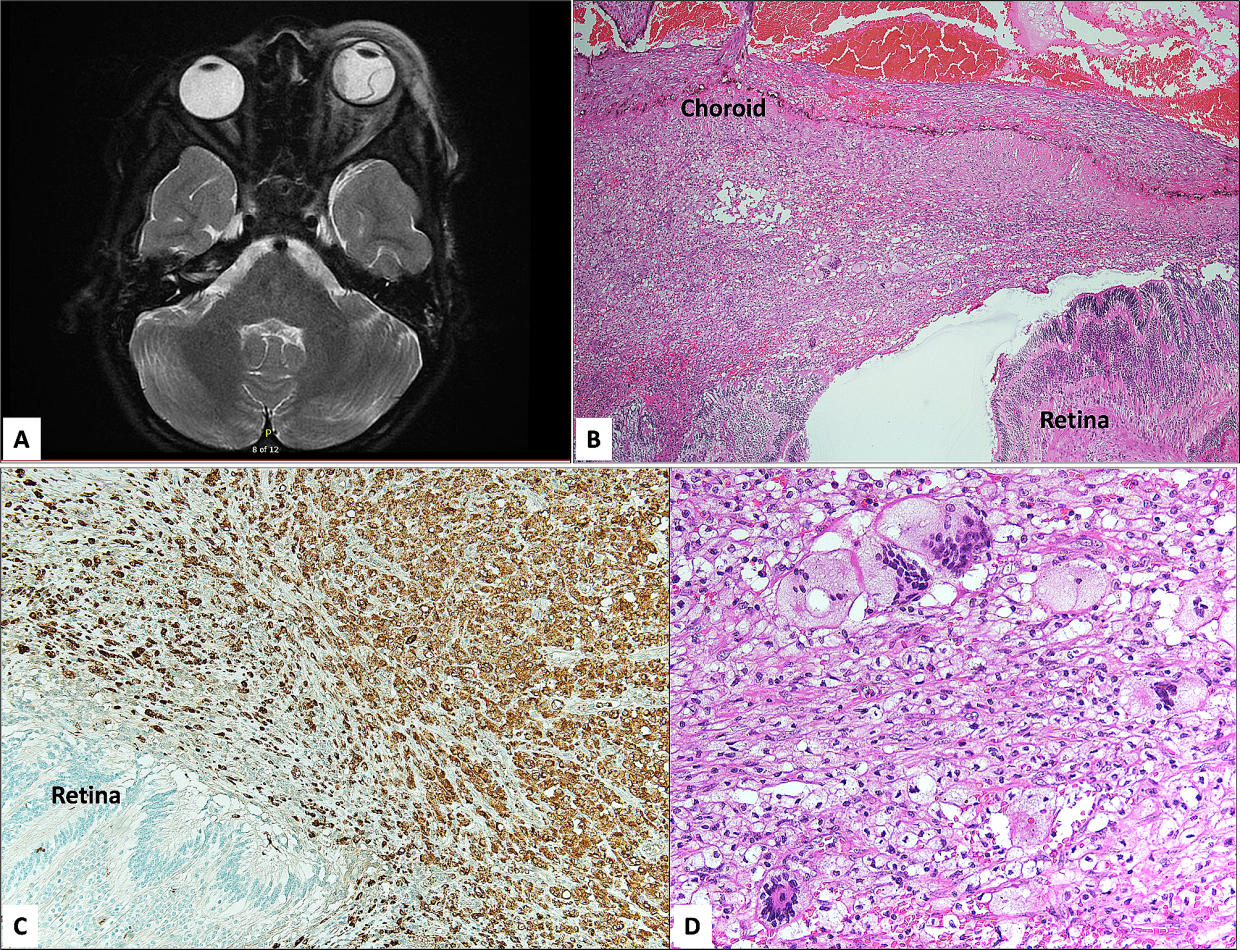
**A**: Axial T2-weighted magnetic resonance image with contrast in the patient with choroidal juvenile xanthogranuloma showing intraocular heterogeneous hypo-intensity of the left globe with thickening of the periocular soft tissues, and proptosis, suggestive of an atypical infection or a neoplastic process. **B**: Histopathological photo of the enucleated left globe showing disorganized thickened choroid and adjacent detached retina (Original magnification x 40 Hematoxylin and eosin). **C**: The globe showing histiocytic infiltrate extending to the subretinal space and merging with the choroid. The numerous lipid-laden histiocytes failed to express S-100, Factor XIIIa, and langerin but were expressing reaction to macrophage marker (Original magnification x 100 CD68). **D**: Numerous atypical Touton giant cells and occasional eosinophils within the infiltrate were seen in this lesion (Original magnification x 200 Hematoxylin and eosin)

The patient with isolated choroidal JXG presented with left eye vision loss, pain, periorbital swelling, proptosis, and a 20% limitation of abduction at the age of 6 years old. Ultrasound Biomicroscopy (UBM) scan demonstrated total closed-funnel retinal detachment with dense vitreous and subretinal opacities in addition to intraocular lesion with diffuse ocular wall thickening by Magnetic Resonance Imaging (MRI) (Fig. 2A). A provisional diagnosis of retinoblastoma with orbital cellulitis was made, for which enucleation was performed (Fig. 2B). The vitreous cavity was heavily infiltrated by foamy histiocytic cells and numerous atypical Touton giant cells (Fig. 2C and D).

The patient who had primary orbital involvement, had associated eyelid lesions but presented with bilateral 3 mm proptosis and inferonasal dystopia. Orbital imaging in that patient showed bilateral ill-defined soft tissue infiltration in the extraconal space, which involved the lacrimal gland in addition to pansinusitis.

Adult xanthogranuloma was found in 8 patients with an age range of 26-60.9 years and a mean age of 44.3 years. The clinical findings in these cases are summarized in Table 3.

**Table 3:** Demographics and clinical presentation of 8 patients with ophthalmic AXG

Case	Age range	Gender	Clinical Presentation		Duration of Symptoms	Treatment
**Eyelid lesions**						
1	50-55	Female	Bilateral Upper and lower eyelid large yellow lesions resembling xanthelasma		13 months	Surgical excision with full thickness skin graft
2	60-65	Male	Right lower lid 8 × 9 mm nodular lesion with central ulceration, madarosis, and orbital involvement		1 week	Surgical excision alone after multiple reoccurrences
3	40-45	Male	Bilateral Upper and Lower yellow nodules		7 years	Surgical excision with full thickness skin graft + intralesional steroids/Avastin + Methotrexate treatment
4	40-45	Female	Right lower eyelid margin 3 × 4 mm globular firm mass attached to the tarsus		3 months	Surgical Excision alone
**Conjunctival/Limbal lesions**						
1	35-40	Male	Right sub-conjunctival 3 × 3 mm yellow firm non-mobile nodule		24 weeks	Surgical excision alone
2	40-45	Male	Right corneal vascularized, keratinized lesion with anterior staphyloma involving entire cornea		2.6 years	Surgical excision + PKP for corneal scarring
3	30-35	Female	Right limbal 4 × 3 mm elevated orange mass at supero-nasal cornea invading all layers of the stroma		8 months	Surgical excision + AMT
4	25-30	Male	Right supero-nasal limbal 9 × 6 mm orange mass with vascularization		3 weeks	Surgical excision + AMT

PKP: Penetrating keratoplasty; AMT: Amniotic membrane transplant

**Fig. 3:**
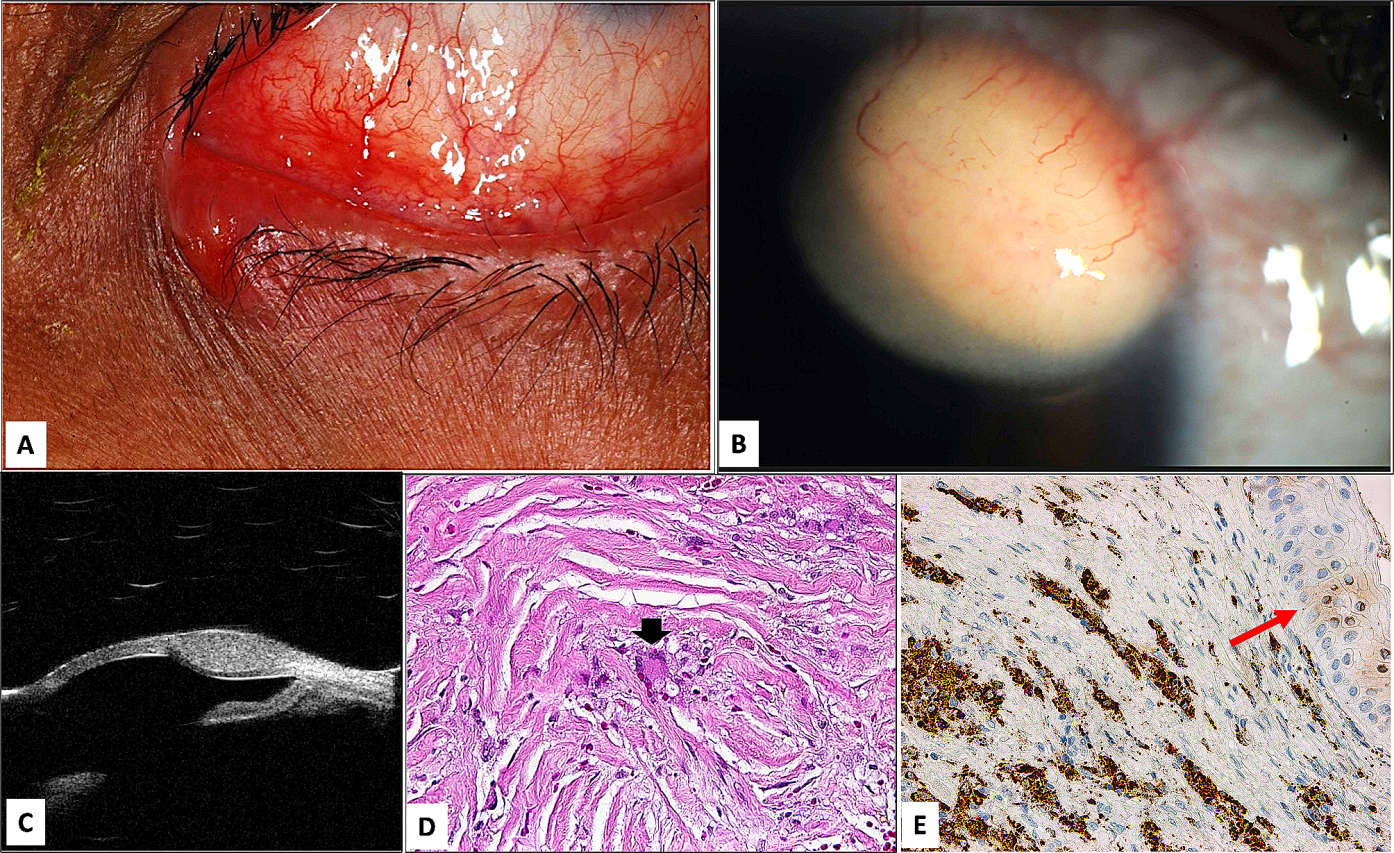
**A**: The clinical appearance of the adult xanthogranuloma (AXG) right lower eyelid nodular lesion with central ulceration in a male, clinically diagnosed as sebaceous carcinoma (Case 2). **B** & **C**: The clinical appearance in a female with right limbal 4.5 × 3.5 mm elevated orange mass supero-nasally in B. The mass is occupying the corneal stroma and pressing over Descemet’s membrane by ultrastructural Biomicroscopy (UBM) in C. **D**: The limbal lesions consisted of sub-epithelial numerous foamy histiocytes and few multinucleated Touton giant cells (red arrowhead) in the AXG lesion (Original magnification x 400 Hematoxylin and eosin). **E**: The stromal infiltrate below the corneal epithelium (red arrow) showing the histiocytic infiltrate expected positivity (Original magnification x 200 CD-68)

Eyelid AXG lesions were variable in size raised, yellowish, and painless. The mean duration of symptoms was 7.3 months (1 week-2.6 years). The clinical provisional diagnosis in this group was xanthelasma (mainly in 2 patients who also had dyslipidemia), and sebaceous carcinoma in 1patient (Fig. 3A). The xanthoma cells showed positive staining with CD68 in all patients while they were negative to S-100 and CD1a.

**Fig. 4:**
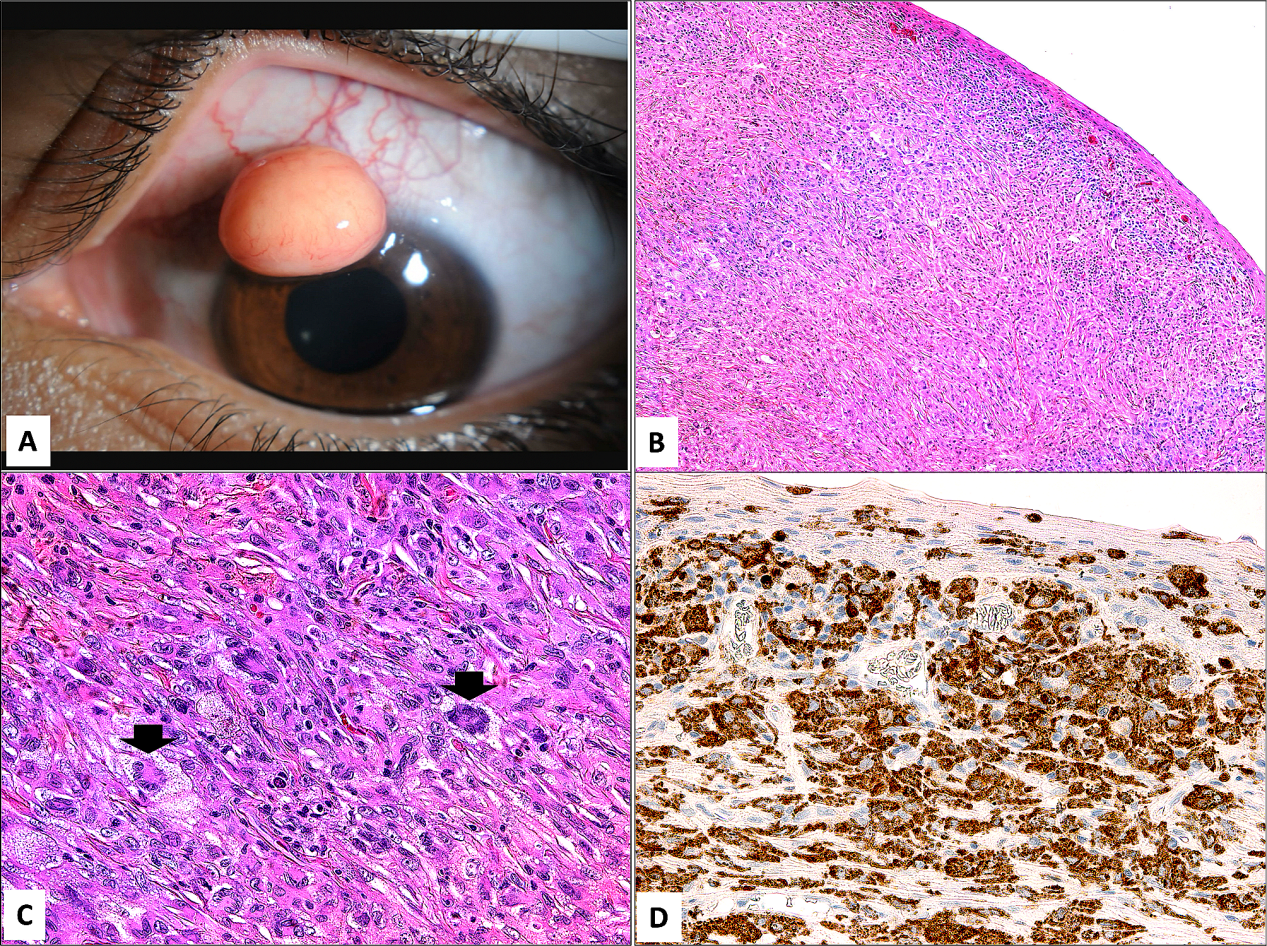
**A**: The clinical appearance of another smaller limbal AXG orange lesion supero-nasally in a young male (Case 2). **B** & **C**: The histopathological typical appearance of XG with sub-epithelial numerous foamy histiocytes and Touton giant cells (black arrowheads) (Original magnification x 100 in B and 400 in C, Hematoxylin and eosin). **D**: The stromal infiltrate showing similar expression of the histiocytes to non-Langerhans’ cells marker (Original magnification x 400 CD-68)

Ocular surface AXG lesions were seen in 4 patients (3 males and 1 female). Their vision ranged from 20/20 to light perception depending on the amount of visual axis obstruction. The pre-operative clinical diagnosis included corneal lipoma and pyogenic granuloma. UBM was done in the female patient (case 3) to evaluate the depth and extent of the lesion (Fig. 3B & C). The limbal lesions had similar histopathological features (Fig. 3D & E) and (Fig. 4A through D).

Successful surgical excision was performed for all conjunctival and limbal lesions without complications, two patients needed an amniotic membrane patch to cover the defect and subsequent PKP was needed in one to treat post-excision corneal scarring.

One patient presented at the age of 11 months with right sub-conjunctival whitish vascular lesion that was extending to the corneal limbus following finger trauma. This patient was diagnosed histopathologically as a case of SRH based on the presence of typical non-Langerhans histiocytic cells with “glassy” appearance and lipid accumulation.

## Discussion

The Xanthogranuloma family includes several entities. They are defined by the age of presentation, focality (solitary, multiple, or generalized), and the organ involved [11]. JXG is by far the most common non-LCH that typically affects the skin as observed in our study [4, 12]. The eye is the most common extracutaneous site of JXG, but other organs such as the brain, lungs, and spleen can also be involved [5, 6]. 

JXG generally appears during the first year of life and the exact incidence is unknown [13]. JXG was diagnosed in 129 out of 24,600 pediatric tumors (0.5%) over 35 years in a large tumor registry [14]. This figure may underestimate the real frequency of JXG as some cases are diagnosed clinically without histopathological confirmation. It has been estimated that 40–70% of non-congenital JXG occur during infancy [4]. The reported median ages of onset in 2 large case series, were 5 months and 12 months, respectively [11, 14]. The male to female ratio was 1.4:1 [14]. In our series, the mean age in the JXG group was 4.5 years, which is much older and the male to female ratio was 1:1.2.

The eyes were variably involved with ocular lesions in about 0.3–10% of children with cutaneous JXG [7, 15]. Ophthalmic involvement was reported in the eyelid, orbit, iris, retina, choroid, and optic nerve [16–24]. JXG of the iris was first reported in 1949 by Blank et al. [25] They are often clinically misdiagnosed as malignant intraocular tumors, which was witnessed in our choroidal JXG case [16]. In 2 series of ophthalmic JXG, the most affected site was the iris [17, 26]. In this current series however, eyelid involvement was the most common in about 2 thirds of the patients (5/11) followed by other sites including the iris and conjunctival/limbal in 2/11 patients each. Secondary glaucoma, requiring anti-glaucoma drops has been reported by Samara et al. in 5 out of their 19 patients with iris JXG [26]. Similarly, one of our patients with iris JXG developed glaucoma.

JXG has been reported to occur in 5–10% of patients with neurofibromatosis type 1 (NF1) and reaching up to approximately 30% [27, 28]. Moreover, it has been suggested that the presence of JXG in young children with café au lait macules is a marker of NF1 even when other diagnostic signs of NF1 are absent [29]. A triad of JXG, NF1, and juvenile myelomonocytic leukemia (a rare form of childhood leukemia) has also been described, which were not detected in our cases [30, 31]. 

The histopathological appearance of JXG is affected by the age of the lesion [32]. The Touton giant cells and the inflammatory cell infiltration are less frequent in intraocular JXG when compared to skin JXG lesions [32, 33] However, the immunohistochemical characteristics remain the same [11]. 

The management of JXG depends primarily on the site(s) of involvement. Involution generally occurs 1 to 5 years after the onset of the lesions leaving, occasionally, atrophic or hyperpigmented scars [34, 35]. For patients with large lesions that are vision-threatening, excisional biopsy is warranted [36]. However, in cases with astigmatic amblyopia, astigmatism may not be fully eliminated after surgical resection of the tumor [36]. 

Orbital lesions are rare with limited publications regarding recommended treatment such as systemic corticosteroid administration or surgical excision [37]. 

Different modalities have been used to manage conjunctival/corneoscleral JXG including steroids (topical, subconjunctival, systemic), excision with cryotherapy and/or topical steroids, and radiotherapy [24, 38–42]. Samara et al. presented 6 eyes with conjunctival JXG treated either by topical steroids, excisional biopsy, or observation [26]. All patients had complete resolution with no recurrence at a median follow-up of 15 months [26]. Ashkenazy et al. reported successful treatment with intravitreal and intracameral bevacizumab (1.25 mg/0.05 ml) for 2 patients presenting with JXG of the iris and the episclera, respectively [43]. Our cases were treated by surgical excisional biopsy and did not show tumor recurrence over follow-up durations between 6 and 24 months.

Frequent topical steroid with slow tapering is the standard treatment for JXG of the iris [26]. Periocular steroids, systemic steroids, or even low dose radiotherapy can be considered if the lesions continued to show insufficient response [7, 44, 45]. Despite treatment of our 2 patients with frequent topical steroids and cycloplegic drops, recurrent hyphema was noted over the subsequent first 4 weeks in both patients.

JXG involving the choroid is rare. In a case series published in 1960, 15 out of 20 eyes with intraocular JXG were enucleated for a suspected malignant intraocular tumor in 13 eyes and secondary glaucoma in 2 eyes [16]. In another more recent series, one patient presented with bilateral unifocal choroidal lesions presumed to be JXG and the use of fine-needle aspiration biopsy was suggested in suspicious cases to prevent unnecessary enucleation [26]. Our patient was treated with enucleation for suspected retinoblastoma in a blind eye and did not show any local recurrence or systemic manifestations for approximately 4 years of follow-up.

Adult xanthogranuloma is the least common among adult orbital xanthogranulomatous diseases [46, 47]. The pathogenesis of AXG was suggested to be related to physical trauma, infection, and hematologic malignancy particularly in patients with multiple AXG [4, 48]. 

The clinical and histopathological features are not statistically different between juvenile xanthogranulomas and adult xanthogranulomas, other than spontaneous regression which was more likely in JXG than AXG [49]. 

The histopathological findings of the lesions in both groups in our series were almost identical; however, our sample size is small to compare the clinical and systemic manifestations.

Adult xanthogranuloma of the orbit and ocular adnexa is rare and typically isolated [50]. The mean age of affected patients is 50 years with no significant gender preference [47, 50]. In our series the mean age was a bit younger (44.3 years) possibly because we have combined our 4 eyelid/orbit cases with the 4-ocular surface cases. However, when the mean ages in these cases were calculated for each of the 2 groups separately, the mean age was 50.5, and 35.5 respectively.

Corneo-limbal AXG is also very rare and occurs primarily in males [11, 51]. Primary AXG of the conjunctiva has also been reported and we had one case in our series [38, 52]. The previously reported cases of limbal AXG in patients who are 18 years of age, or more are summarized in Table 4 including our previously published case [12]. The calculated mean age of onset across reports of limbal adult xanthogranulomas is 34.5 years (and 35.5 years in this current study), which is younger than the reported average age for eyelid/orbital AXG group.

**Table 4:** Summary of all adult corneo-limbal AXG cases (Age ≥ 18 years) reported in the English-written literature

Case	Year of publication	Source	Age*	Gender	Extraocular lesions
1	1984	Collum et al. [53]	18	Male	No
2	1994	Harvey et al. [51]	30	Male	No
3	2001	Wang et al. [54]	45	Male	No
4	2002	Mohamed et al. [55]	39	Male	Yes (face, trunk, axilla, and groin)
5	2002	Kobayashi et al. [56]	40	Female	No
6	2007	Hirata et al. [57]	48	Female	No
7	2009	Soler et al. [58]	18	Male	No
8	2010	Hermel et al. [59]	35	Male	Not reported
9	2011	Callejo et al. [60]	33	Male	Not reported
10	2014	Castro-Gómez et al. [61]	25	Female	No
11	2014	Kontos et al. [62]	67	Male	No
12	2016	Alkatan et al. [12]	43	Male	No

*Calculated average age is 34.5 years

Clinical appearance of the eyelid lesions in AXG has been variable [63, 64]. AXG can also present atypically as an isolated eyelid margin papillary mass similar to our second case [65]. It is worth mentioning that 2 cases from our series have been previously reported, one eyelid/orbital and one limbal [12, 66]. Patients with xanthogranulomatous lesions of the corneo-limbus may complain of foreign body sensation or the cosmetic appearance of the progressively enlarging lesions [67, 68]. The lesion is described to be more yellowish with time suggesting a progressive lipid deposition as the lesion matures [56, 59]. However, AXG of the limbus can be aggressive with severe inflammation and corneal stromal infiltration as observed in our female patient [62]. 

AXG has been linked to IgG4-related disease in which increased numbers of IgG4 + plasma cells are characteristic [8, 69]. In our series 2 out 8 cases have been tested for the presence of abnormal levels of IgG4 + cells. None of the AXG patients in our series showed confirmed systemic manifestation of IgG4-RD.

Treatments deployed for the treatment of xanthogranulomatous diseases include surgery, local and systemic steroids, methotrexate, radiation, cyclosporine, and other immunosuppressive agents [47, 70–74]. Surgical excision is the mainstay treatment for corneo-limbal AXG, despite the use of short course of topical steroids by some [58]. If the deeper corneal stroma is involved or the lesion is recurrent, lamellar keratoplasty may be appropriate [53, 56, 59]. 

Solitary reticulohistiocytoma (SRH) is another rare, benign histiocytic proliferation with predominance of oncocytic macrophages and ground-glass giant cells. Our single patient has been previously reported as an individual case report, thus was not further discussed in this series [75]. 

## Conclusions

Ophthalmic mucocutaneous non-LCHs disease has variable manifestations mostly eyelid lesions in about half of the patients, ocular surface masses in about one-third, followed by iris involvement in a smaller number of cases, orbital involvement, and finally intraocular (choroidal) involvement, which is extremely rare. Histopathological evaluation is required for definitive diagnosis. JXG was the most common disease entity in the current series with a relatively older age (average of 4.5years) compared to previously published series. Given the rarity of the disease, ophthalmic JXG should be included in the differential diagnoses of eyelid, orbital, ocular surface, and intraocular tumors in the pediatric population. AXG presents at an older age -as expected- with an average age at presentation of 44.3 years. Ocular surface AXG tends to occur at a younger age. Surgical intervention was the most common treatment instituted to our patients. Future research on molecular genetics in relation to the pathogenesis of histiocytic disorders may permit more specific and selective therapies.

## Data Availability

The datasets used and/or analyzed during the current study are available from the corresponding author on reasonable request.

## Abbreviations

AXG: Adult xanthogranuloma
HEC/IRB: Human Ethics Committee/Institutional Review Board
IgG4-RD: IgG4-related disease
IgG4-ROD: IgG4-related ophthalmic disease
JXG: Juvenile xanthogranuloma
KAUH: King Abdulaziz University Hospital
KKESH: King Khaled Eye Specialist Hospital
LCH: Langerhans cell histiocytosis
MRI: Magnetic resonance imaging
SRH: Solitary reticulohistiocytoma
UBM: Ultrasound Biomicroscopy
